# Subretinal Injection Volume Correlates to Persistent Outer Retinal Thinning in the Pig Eye

**DOI:** 10.1167/iovs.66.14.27

**Published:** 2025-11-13

**Authors:** Immanuel P. Seitz, Ruslan Nyshchuk, Zdenka Ellederova, Taras Ardan, Jana Juhasova, Yaroslav Nemesh, Stefan Juhas, Saskia Drutovic, Daniela Süsskind, Sven Schnichels, Jan Motlik, Nikolai Klymiuk, Uwe Wolfrum, Kathryn Post DeMott, Jennifer J. Klein, Ashley Winslow, M. Dominik Fischer

**Affiliations:** 1 Oxford Eye Hospital, Oxford University Hospitals NHS Foundation Trust, Oxford, United Kingdom; 2 Nuffield Department of Clinical Neurosciences, University of Oxford, Oxford, United Kingdom; 3 University Eye Hospital Tübingen, Eberhard-Karls-Universität Tübingen, Tübingen, Germany; 4 Institute of Animal Physiology and Genetics, Czech Academy of Sciences, Prague, Czech Republic; 5 Department of Cell Biology, Faculty of Science, Charles University, Prague, Czech Republic; 6 Medical Department I, Cardiology, Angiology, Pneumology, Klinikum rechts der Isar, Technische Universität München, München, Bayern, Germany; 7 Center for Innovative Medical Models, Ludwig-Maximilians-Universität München, München, Germany; 8 Institute for Zoology, Department I, Johannes-Gutenberg-Universität, Mainz, Germany; 9 Institute of Quantitative and Computational Biosciences (IQCB), Johannes Gutenberg-University Mainz, Mainz, Germany; 10 Odylia Therapeutics, Atlanta, Georgia, United States

**Keywords:** AAV, gene therapy, retinal thinning, atrophy, subretinal injection, porcine, pig

## Abstract

**Purpose:**

Transgenic pig models are a valuable model for preclinical testing of gene and cell therapies. Subretinal injection (SRI) is a common drug delivery method but has been associated with retinal thinning and atrophy. This study examined whether SRI volume correlates with retinal thinning in the pig eye and compared the effects of balanced salt solution (BSS) and adeno-associated virus (AAV) injections.

**Methods:**

Ten eyes from five transgenic pigs were included in this study. Eight eyes received escalating subretinal injection volumes (SRVs) (50, 100, 150, and 200 µL) of either BSS or AAV [5 × 10^11^ vg/mL], targeting the cone-rich area centralis of the pig eye. After six months, outer retinal thickness inside versus outside the bleb area (ΔORT) was quantified using optical coherence tomography (OCT). Histology was performed to confirm OCT findings.

**Results:**

Treated eyes showed clinically relevant (−21.5 ± 2.7 µm) outer retinal thinning inside the bleb area (*P* = 0.0001). A strong, statistically significant, linear correlation (*R*² = 0.73 *P* = 0.0068) was found between SRV and ORT loss. There was a 1 µm loss of ORT for every 9 µL of SRV. ORT loss was similar between AAV and BSS, except at the highest volume (200 µL, 1 × 10^11^ vg), where greater thinning occurred with AAV over BSS (Δ11 µm).

**Conclusions:**

This study supports the notion that SRV could be an independent factor in development of outer retinal thinning in the pig eye. Modifying surgical technique to favor the placement of multiple smaller blebs of <100 µL might mitigate retinal thinning because of volumetric stress and enhance the preclinical safety profile of investigational therapies.

Pig models are a promising avenue for preclinical testing of novel gene and cell therapies in the eye. Pig eyes are very similar in size to humans,^1^ which makes them ideal for establishment and optimization of novel surgical techniques in the eye.^2^^–^^4^ Like primates, and in contrast to rod-dominated murine models,^5^ the pig retina also has plenty of cones^6^ enabling study of cone-centric disease. The pig's cone-rich area centralis is the visual streak, a sparsely vascularized band of high cone density that traverses the central retina along a horizontal meridian, from temporal to nasal.^7^ Furthermore, the pig is amenable to genetic modification via CRISPR and somatic cell nuclear transfer.^8^ It’s high fertility (two or three litters per year) and large litter size (on average 12 piglets)^9^ facilitate expansion of transgenic offspring and enable highly standardized same-litter experimental designs, which would not be practical with other large animal models, such as nonhuman primates. Transgenic retinal disease models in the pig include various mutants in *RHO*, namely P347L^10^^,^^11^ and P23H,^12^^,^^13^ as well as *GUCY2D,*^14^ *USH1C*,^8^ and *ABCA4*.^15^ These can and have been used to evaluate novel therapeutic approaches, such as AAV-based gene therapy^15^^,^^16^ and stem cell therapy.^17^^–^^20^

These preclinical studies often rely on subretinal injection (SRI) to deliver viral vectors or stem cells. SRI provides excellent access to photoreceptors and retinal pigment epithelium. However, it involves an inherently traumatic detachment of the neuroretina to create a subretinal fluid compartment (“bleb”). To limit the surgical sequelae of SRI, the technique has undergone continuous refinement in human ophthalmic surgery,^21^^,^^22^ aided by innovations such as automated, pressure-controlled delivery systems, and modified surgical protocols.^23^^,^^24^ In contrast, optimization of SRI for the anatomical and biomechanical particularities of the pig retina is a fairly recent endeavor.^25^^–^^27^ One fundamental parameter of SRI that remains poorly defined in the pig eye is the subretinal volume (SRV) that can safely be introduced into the pig subretinal space. An independent effect of SRV on post-surgical retinal thinning is plausible, as SRV is the main modifier of shearing forces^22^ and retinal tension^28^^,^^29^ during SRI and could therefore mediate damage to both the inner and outer retina. Although studies have reported in detail on the influence of injection pressure on morphological outcomes in the pig eye,^27^ no direct dose–response relationship between SRV and tissue loss has been established so far for this valuable large animal model. Characterization of SRV in the pig retina is essential, because retinal thinning and atrophy due to volumetric load alone could be a critical confounder when investigating the safety and tolerability of novel therapies delivered via SRI. Therefore this study aimed to investigate the relationship between SRV and the severity of postoperative retinal thinning in the pig eye to ultimately identify a safe upper limit for SRV in the porcine eye. In addition, SRI was performed using either balanced salt solution (BSS) or adeno-associated virus (AAV) to probe for a potential synergistic interaction of pro-atrophic factors in the development of retinal thinning and atrophy.

## Methods

**Figure 1.**
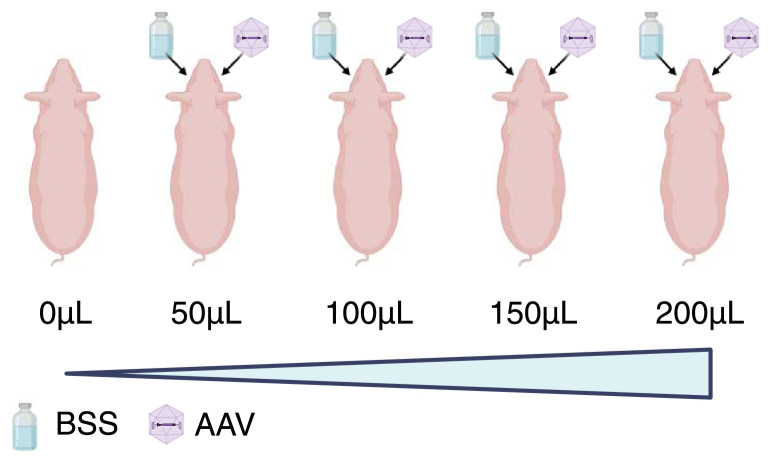
**Experimental setup.** Four animals homozygous for mutations in *USH1C* from the same litter were treated with a single subretinal injection of either BSS (OS) or AAV (OD). The subretinal injection volume per eye was escalated from one animal to the next (0–200 µL in 50 µL increments). Created in https://BioRender.com by Sven Schnichels (license holder: José Hurst, Schnichels Lab), 2025, https://BioRender.com/qrb0wrw, agreement no. BE28A0JQBE.

Animal experiments were approved by the State Veterinary Administration of the Czech Republic and performed at the PIGMOD Centre, Institute of Animal Physiology and Genetics of the Czech Academy of Sciences, under the experimental protocol number 75/2019. The procedures were in adherence to the ARVO Statement for the Use of Animals in Ophthalmic and Vision Research. Five transgenic midipigs from the same litter, two males and three females, were included in this study. They were homozygous for the c.C91T/p.R31X mutation in the *USH1C* gene. Age at intervention was three months, with a body weight of approximately 30 kg. One animal served as an untreated control. Four animals underwent 23G pars plana vitrectomy combined with subretinal injection. Treated animals received escalating subretinal injection volumes (50, 100, 150, and 200 µL) of either BSS (OD) or AAV (OS) as shown in Figure 1.

### Ocular Surgery

General anesthesia was induced by intramuscular injection of 2mg/kg azaperone (Elanco Colombia s.a.s, Bogota, Colombia), 8 µg/kg atropine (Roche, Basel, Switzerland), and 20 mg/kg ketamine (Bioveta, Ivanovice na Hané, Czech Republic)). Atracurium 0.67 mg/kg (Kalceks, Riga, Latvia) was given before intubation, and anesthesia was maintained with continuous propofol perfusion, 1% at 20 mL/h (Fresenius Kabi Deutschland GmbH, Bad Homburg, Germany)). Before surgery, the periorbital regions were thoroughly cleaned with povidone iodine (EGIS, Budapest, Hungary), and sterile surgical drapes (Hartmann, Heidenheim, Germany) were applied. A lid speculum was then placed, and three sterile small-gauge trocars (23G; DORC, Zuidlands, Netherlands) were inserted through the pars plana. Core vitrectomy was performed using an ophthalmic surgery microscope (Haag-Streit Surgical, Wedel, Germany), vitrectomy machine (Ruck GmbH, Eschweiler, Germany), and vitrector (Optikon, Rome, Italy). After vitrectomy 50, 100, 150, or 200 µL were applied to the subretinal space, targeting the visual streak (VS) BSS (Zeiss, Oberkochen, Germany) or injectable AAV was delivered into the subretinal space via a handheld 41G extendable cannula (1270.EXT; DORC). The vector used, AAVanc80.CAG.USH1C_a1 (OT_USH_101), has been published previously.^16^ The same vector concentration of 5 × 10^11^ vector genomes (vg) per mL was applied regardless of SRV. Therefore the vector dose (vg/eye) increased stepwise from 2.5 × 10^10^ at 50 µL, to 5 × 10^10^ at 100 µL, 7.5 × 10^10^ at 150 µL, and 1 × 10^11^ at 200 µL. All surgeries were performed by a single experienced surgeon to ensure consistency across groups. After the subretinal injection, the trocars were removed, and a 0.05 mL solution containing 1 mg dexamethasone (Organon, Jersey City, NJ, USA) was applied under the conjunctiva, and 0.3% ofloxacin ointment (Bausch & Lomb, Rochester, NY, USA) was applied to the surface of the eye before the animal's recovery. For postoperative care, animals in both groups received 0.5% moxifloxacin (Novartis, Basel, Switzerland), and 1% prednisolone eye drops (Ursapharm, Saarbrücken, Germany) three times daily for a week. Systemic immunosuppression included prednisone at 1 mg/kg intramuscular (Zentiva, Prague, Czech Republic) from day 2 until day 5, followed by 0.5 mg/kg for the next seven days, and then 0.25 mg/kg for another seven days.

### Optical Coherence Tomography

**Figure 2.**
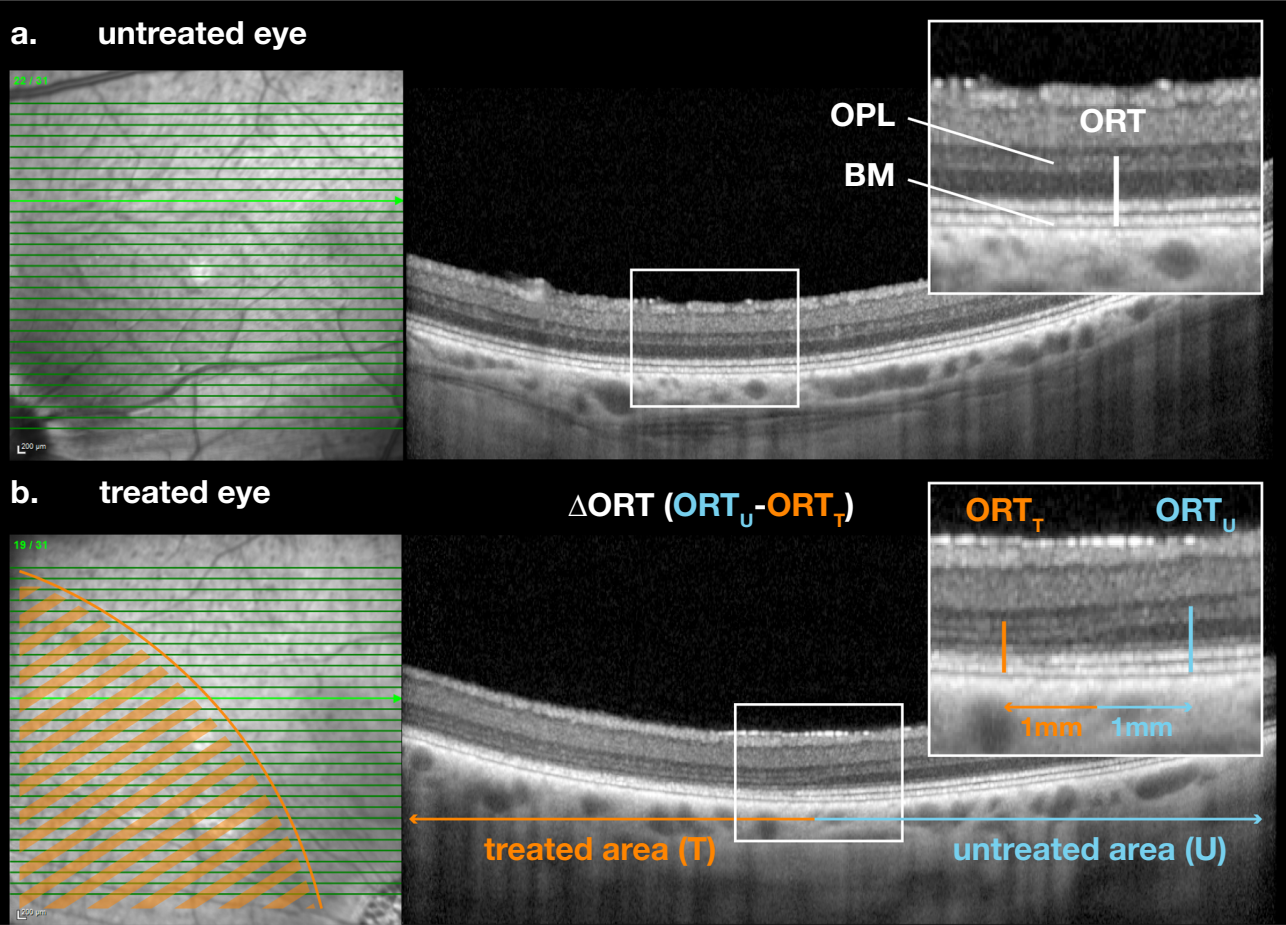
**Quantification of ORT on OCT. Comparison between treated and untreated area. *Left*: Infrared (IR); Right: corresponding OCT.** **(a)** Within the visual streak, untreated eyes demonstrated uniform ORT along horizontal meridians, defined as the distance between the outer plexiform layer (OPL) and the basement membrane (BM). **(b)** In treated eyes there was a loss of ORT, which was strictly limited to the treated area. ORT was compared between treated (*T*) and untreated (*U*) areas. For this, the bleb border was identified, and ORT thickness measurements were taken 1 mm toward the treated (inside bleb, *ORT_T_*) and untreated (outside bleb, *ORT_U_*) area. From this, the loss of ORT (ΔORT = ORT_U_ − ORT_T_) because of treatment was determined. *Orange overlay*: treated area, inside bleb; *blue overlay*: untreated area, outside bleb.

Six months after treatment all animals underwent examination including optical coherence tomography (OCT) and fundus autofluorescence (AF-SLO) using a Heidelberg Spectralis Flex OCT device (Heidelberg Engineering, Heidelberg, Germany). Total retinal thickness (TRT) and outer retinal thickness (ORT) were quantified using OCT. ORT was defined as the distance between the outer plexiform layer and the basement membrane. First, the border of the subretinal bleb was first identified using infrared AF-SLO and surgical video footage. Measurements of ORT were then taken from horizontal B-scans at 1 mm to either side of the bleb border (i.e., both toward the treated area [inside the bleb] and the untreated area [outside the bleb]). The difference between these values was used to assess the degree of outer retinal thinning (ΔORT) attributable to the treatment (Fig. 2). Measurements were performed by a masked observer in triplicate. In untreated eyes (no bleb border), TRT and ORT were measured at predefined positions along multiple horizontal (1, 2, and 3 mm nasal to the optic nerve head) and vertical (3, 4, and 5 mm superior to the optic nerve head) meridians to rule out confounding variations of retinal thickness because of anatomy alone.

### Histology

After sacrifice, eyes were enucleated, fixed in 4% paraformaldehyde and stored at 4° C. Eyes were opened in anteroposterior direction through the pupil and optic nerve resulting in a nasal and temporal half. The calottes were then dehydrated through an increasing alcohol series, then xylol and finally embedded in paraffin using the Tissue Processing Center (MEDITE Medical GmbH, Burgdorf, Germany). Tissues were sectioned into 5 µm thick specimens using a microtome (Leica RM2245; Leica Biosystems, Wetzlar, Germany) with subsequent dewaxing and hematoxylin and eosin staining. Semiquantitative assessment of the section was performed in a masked fashion, scoring (0 = none; 1 = minimal; 2 = slight; 3 = moderate; 4 = severe) the following parameters: retinal atrophy, retinal detachment, retinal vacuolation, retinal (peri)vasculitis, choroidal (peri)vasculitis, inflammatory cells (choroid), inflammatory cells (retina), hemorrhages (choroid), hemorrhages (retina), and irregular pigmentation.

### Statistical Analysis

Data analysis was carried out at University of Oxford, UK. Statistical analysis was performed using JMP 18.0 (SAS Institute Inc, Cary, NC, USA). Because of the overall small sample size, all data were tested for normality using the Shapiro-Wilk W test to select appropriate statistical tests. Student's *t*-test and one-way ANOVA were used to compare group means for normally distributed data (e.g., AAV- vs. BSS-treated eyes). For paired samples (e.g., ORT inside vs. outside bleb) paired-sample tests were performed. For subgroup analyses that did not satisfy normality criteria because of small sample size (i.e., by injection volume *and* treatment), the data was analysed using nonparametric tests or descriptive statistics only. Linear regression results are reported using non-adjusted Pearson *R*^2^, the corresponding *P* value, and the slope *m* of the linear fit line. All values are reported as mean ± SD unless otherwise specified.

## Results

### ORT in the Visual Streak Shows Virtually No Natural Variability Along Horizontal Meridians

Total retinal thickness in the visual streak was found to vary significantly along vertical meridians (*P* < 0.0001, Supplementary Fig. S1a). There was no significant variability of TRT along horizontal meridians (ns, Supplementary Fig. S1b). However, the absolute values along each horizontal meridian were heavily dependent on the vertical position of the scan, resulting in larger error margins. ORT showed a highly homogenous thickness along both vertical (ns, Supplementary Fig. S1c) and horizontal (ns, Supplementary Fig. S1d) meridians. In untreated eyes, repeated measurements at 2 mm distance along horizontal meridians in the visual streak (e.g., 1 vs. 3 mm nasal to optic nerve head) incurred virtually no change in measured ORT (i.e., 116 ± 0.5 vs. 117 ± 1.5 µm). These results confirmed ΔORT along the horizontal meridian as the endpoint of choice for comparing the outer retinal thinning in the treated (inside bleb) versus untreated (outside bleb) areas.

### Persistent ΔORT Inside the Bleb Area Was Observed in All Treated Eyes and Strongly Correlated With SRV

**Figure 3.**
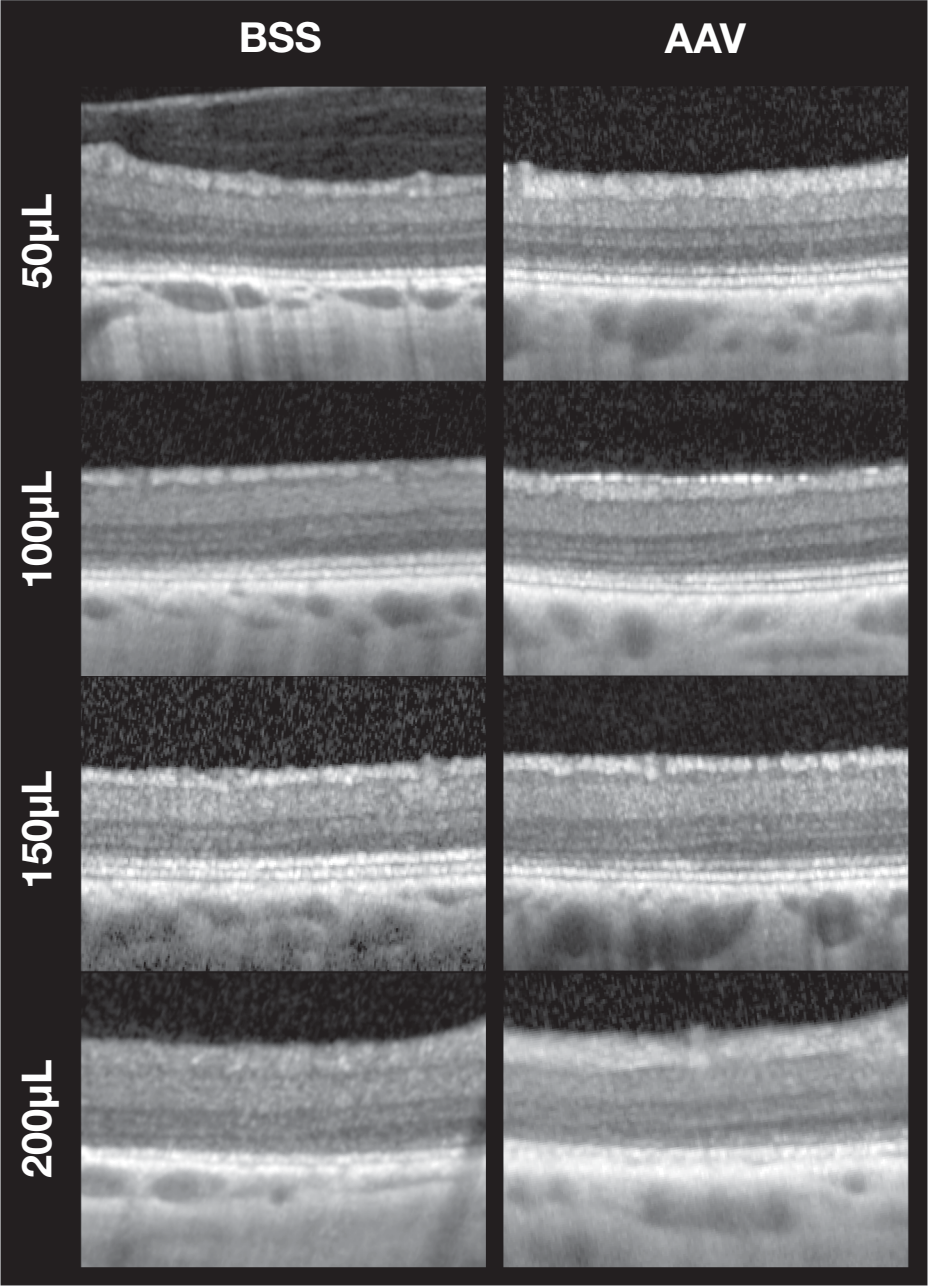
**Qualitative comparison of outer retinal changes across the bleb border on OCT by subretinal (SR) injection volume and treatment (BSS vs. AAV).** These representative OCT scans are centered on the bleb border. Analogous to Figure 2b, the treated area (inside the bleb) is located to the left, the untreated area (outside the bleb) to the right. Eyes treated with 50 µL of either BSS or AAV showed well-preserved ONL with minimal thinning. Injection volumes of 100 to 200 µL all caused marked outer retinal thinning, with partial to subtotal loss of the ONL on OCT. Inner retinal layers were unaffected.

**Figure 4.**
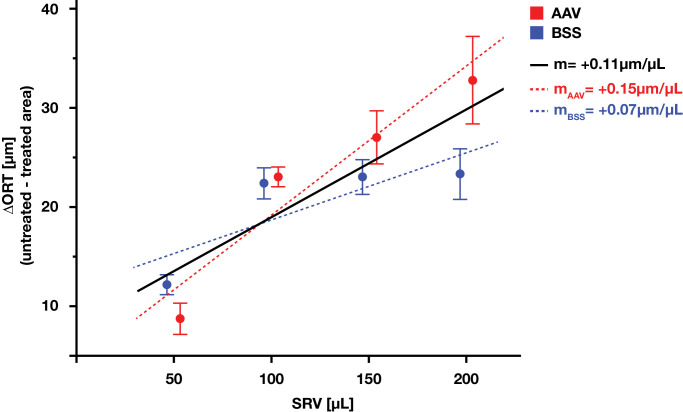
**The ΔORT by SRV. Linear regression.** There was a strong, statistically significant, linear correlation between injection volume and loss of ORT. For every 9 µL of subretinal injection volume there was 1 µm of outer retinal thinning (*R*^2^ = 0.68, *P* < 0.0001). Of note, the linear fit slightly over-estimated thinning at the lowest volume (50 µL). Injection volumes of 100 to 200 µL all caused marked outer retinal thinning. *Dots* and *error bars*: mean ± SD. *Black line*: Linear regression fit line, all eyes pooled. *Dotted lines*: Linear regression fit lines AAV and BSS. *Red*: AAV treatment. *Blue*: BBS treatment.

The ΔORT inside the bleb area was observed in all treated eyes (*P* = 0.0001). ORT inside the bleb area decreased by 21.5±2.7 µm compared to the ORT outside the bleb area. This loss seemed to be driven by reductions in the outer nuclear layer (Fig. 3). There was a strong (*R*^2^ = 0.73) statistically significant (*P* = 0.0068) linear correlation between ΔORT and SRV. Overall, for every 9 µL of injected volume there was 1 µm of outer retinal thinning (Fig. 4) six months after single subretinal injection. SRVs ≥100 µL were associated with pronounced outer retinal thinning of 22.7 ± 0.5 (100 µL) to 28.0 ± 6.6 µm (200 µL). Although likely not entirely without thinning, the lowest SRV of 50 µL induced a loss of just 10.3 ± 2.4 µm, near the lower limit of quantification when using OCT.

### Histology Confirmed Retinal Thinning Driven By Outer Nuclear Layer Loss, Without Overt Signs of Inflammation

**Figure 5.**
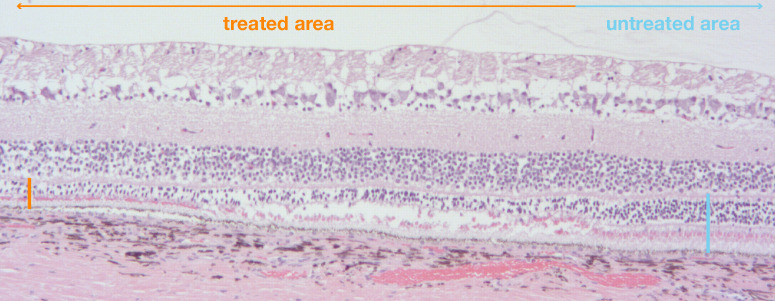
**Histology confirmed persistent outer retinal thinning driven primarily by a reduction of the outer nuclear layer.** As suggested by OCT, the loss of outer retinal thickness seemed primarily driven by a reduction of photoreceptor nuclei in the outer nuclear layer. No inflammatory changes such as retinal (peri)vasculitis, choroidal (peri)vasculitis, inflammatory cells (choroid), inflammatory cells (retina) were noted in any eye six months after treatment. *Orange bar*: ORT in treated area. *Blue bar*: ORT in untreated area.

OCT findings were confirmed on histology, which recapitulated the clear border between the treated and untreated areas, and pronounced outer retinal thinning. As suggested by the OCT, the loss of ORT seemed primarily driven by a reduction of photoreceptor nuclei in the outer nuclear layer. Of note, no inflammatory changes such as retinal (peri)vasculitis, choroidal (peri)vasculitis, inflammatory cells (choroid), inflammatory cells (retina) were noted in any eye, regardless of treatment status (sham or AAV). Figure 5 gives a sample histological section, showing the bleb border region of the eye treated with 150 µL of AAV.

### AAV Over BSS Was not Clearly Associated With Increased Thinning. However, a Synergistic Pro-Atrophic Interaction of AAV and High Injection Volumes Could not be Ruled Out

**Figure 6.**
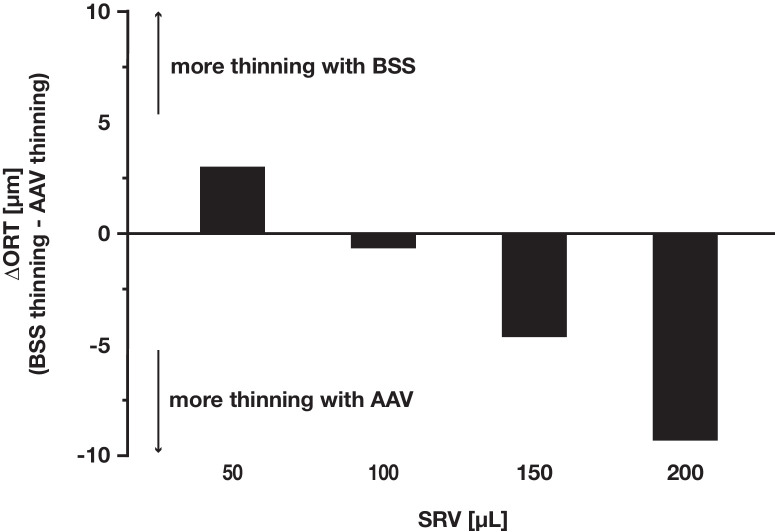
**The ΔORT between AAV versus BSS, by subretinal injection volume.** For higher volumes (e.g., 150 and 200 µL), there was a tendency toward greater thinning in AAV-injected eyes, compared to BSS-injected eyes. The same was not seen in lower volumes (e.g., 50 and 100 µL). Y-axis (ΔORT [µm]): ORT between BSS-treated eyes and AAV-treated eyes. Formula: ΔORT = (BSS thinning) − (AAV thinning).

There was no indication for increased outer retinal thinning when injecting AAV instead of BSS, except when using the largest injection volume of 200 µL (Fig. 6). In a pooled, volume-matched analysis of all eyes, there wasn't a statistically (*P* = 0.67) or clinically significant difference in ORT loss between eyes treated with BSS (20.2 ± 5.5 µm) versus AAV (22.8 ± 10.2 µm). This study was not sufficiently powered to confirm a potential pro-atrophic synergistic interaction of AAV and high volume. Descriptively however, atrophy in BSS-treated eyes seemed to plateau at an SRV ≥100 µL, whereas it continued to climb with higher volume in AAV-treated eyes. This was in line with a very strong sigmoidal fit of the data (*R*^2^ = 0.94), which was primarily driven by BSS-treated eyes. The inflection point of the sigmoidal regression was located at an SRV of 54 mL (*P* = 0.0018), with an upper asymptote of 27.3 µm (*P* < 0.0001) of outer retinal thinning. Linear regression for AAV- and BSS-treated subgroups showed an SRV/ΔORT ratio of 7 µL/µm (AAV) and 14 µL/µm (BSS), respectively.

## Discussion

The pig eye is a promising model for the evaluation of gene and cell therapy and SRI is still the gold standard for drug delivery to the outer retina. So far, the SRV and retinal thinning after SRI is poorly characterized in the pig eye. Therefore this study aimed to investigate a potential relationship between SRV and subsequent retinal thinning. ORT in the pig area centralis, the visual streak, was highly consistent along horizontal meridians. This confirmed that ΔORT over the bleb border (i.e., within vs. without) measured along the horizontal meridian is a robust endpoint for assessing retinal thinning after SRI. Statistically significant and clinically relevant ORT was observed in all treated eyes. Furthermore, there was a statistically significant, strong correlation of SRV and ΔORT. For every 9 µL of injected volume, a 1 µm reduction in ORT was observed six months after treatment. Although volumes of ≥100 µL induced marked outer retinal thinning, the lowest volume of 50 µL induced only minor changes, close to the lower limit of quantification. Looking at the regression analyses for BSS and AAV in isolation, retinal thinning with BSS seemed to plateau at around 100 µL, while for AAV thinning continued to deepen linearly up to the highest volume of 200 µL. Comparison of same-volume injections of AAV versus BSS showed no significant difference in ΔORT, except for the highest SRV (200 µL) and total dose (1 × 1011 vg/eye). Together, these observations could imply that 100 µL represents a volume limiting effect threshold for SRV in the pig eye. Up to 100 µL thinning seemed to be primarily driven by SRV with no evident contribution of AAV. The excess thinning between BBS and AAV at 200 µL could be an expression of either a straight up dose-limiting toxicity related to the escalated vector dose (vg/eye), or of a multifactorial etiology in which both vector-related toxicity and inflammation, as well as mechanical stress and SRV, contribute to thinning of the outer retina.

The observed tolerability of volumes <100 µL for both AAV and BSS is in line with several recent in vivo studies in pigs that have demonstrated good safety profiles for AAV-based GT when applying an SRV of 50 µL.^16^^,^^30^ The notion of ≥100 µL as a volume-limiting barrier in the pig eye is further supported by other recent surgical studies in the pig, in which subretinal injection of 100 µL resulted in outer retinal atrophy.^25^ The observed correlation of SRV and ONL thinning raises the question if the pig eye is uniquely sensitive to subretinal injection trauma and volumetric load, compared to other model species and humans.

In rabbits, subretinal injection of 50 µL of BSS has been shown to cause marked outer retinal degeneration at 28 days after injection, clearly demarcated on OCT and histology.^31^ Two other reports, however, described no such thinning using 50 µL of BSS or conbercept.^32^^,^^33^ This disparity could be the result of a confounding surgical variable (e.g., injection pressure) or a failure to detect existing thinning, which might be noticeable only on higher definition OCT systems. Of note, based on eye size injection of 50 µL in the rabbit is similar to 100 µL in nonhuman primates (NHPs) or 150 µL in the pig or human. In NHPs subretinal sham injections of 100 µL^34^ seem to produce mild changes (i.e., transient disruption of mostly outer segments [OS], with gradual and near-complete structural recovery).^35^ Looking at higher volumes in NHPs, our own group has reported on foveal ONL thickness following subretinal sham injections of 200 µL.^35^ Here, some degree of persistent ONL thinning (instead of OS only) was seen descriptively at 13 weeks after the injection. However, ONL thinning was mild (−7 µm) and not statistically significant compared to intravitreal controls. In addition, although only mentioned as a qualitative finding in the supplementary material, another recent study in NHPs also described thinned photoreceptor layers (i.e., ONL) after injection of 180 µL of formulation buffer.^15^ In dogs, subretinal injection has been associated with retinal lesions and thinning, which were interpreted as traumatic.^36^^–^^38^ From a volume perspective, a study by Annear et al.^39^ was potentially suited to investigate the influence of SRV in dogs: nine animals received subretinal injections of rAAV2.hRPE65p.hRPE65 in both eyes. One eye of each animal was treated with 250 µL, the partner eye with 500 µL. Unfortunately, exact retinal thickness measurements that would enable detection of changes at the 5-20 µm scale were not available. However, tapetal hyperreflectivity, a fundoscopic surrogate for retinal thinning, was seen in 3/9 eyes treated with 500 µL over 1/9 treated with 250 µL. Collectively, the evidence suggests that subretinal injection of larger volumes likely induces a certain degree of ONL thinning in any animal model. However, species-specific differences in susceptibility to SRV appear to exist. Rabbits and pigs may exhibit a more pronounced response to increased SRV relative to NHPs and potentially dogs, although evidence for the latter remains limited.

Humans are not subjected to sham subretinal injections, and treatment volume is usually a fixed variable in both clinical trials and approved GT (i.e., voretigene neparvovec [VN; Luxturna, Spark Therapeutics Inc., Philadelphia, PA, USA]). Therefore isolating a potential adverse effect of SRV alone from published clinical data is challenging. Clearly, the presence of outer retinal thinning observed in this pig study relates closely to the post-approval reality of VN in human patients, because some degree of outer retinal thinning, up to full-thickness (chorio-)retinal atrophy, has emerged as a key side effect after subretinal injection with VN. Looking at the history of VN tolerability through the lens of SRV, it is noteworthy that the preclinical VN studies in RPE65^−^^/−^ Briard dogs^40^^,^^41^ and NHPs^42^ in which no retinal thinning and atrophy were reported used smaller SRVs of 100-200 µL, compared to a larger SRV of 300 µL in human patients.^43^ Certainly, this is a very simplified view that disregards dose, eye size, improvements of surgical technique, and potential species-specific sensitivity to SRV. Regardless, the results of this study indicate that it would be of interest to compare the safety outcomes of treatment with VN between patients treated with one large bleb (∼73%) versus multiple smaller blebs (∼27%).^44^ So far, no clear picture has emerged on whether treatment outcomes in VN correlate to the used SRV.

 This study has several limitations. In most preclinical studies and clinical trials volume is a fixed parameter. Here, AAV “dose” (vg/eye) and AAV “concentration” (vg/mL) are always proportional. In an escalating volume setting however, either AAV dose (vg/eye) or AAV concentration (vg/mL) can be kept constant, but not both. In a *fixed dose* design, the multiplicity of infection (MOI) within the treated retinal tissue decreases with increasing injection volume, as more and more cells are treated with the same absolute number of AAV particles. In a *fixed concentration* design on the other hand, the MOI in the treated area remains stable at the expense of having to escalate the total vg dosage per eye. This study followed a *fixed concentration* design because many dose-limiting concerns such as retinal thinning, chorioretinal atrophy and retinal inflammation are inherently focal (i.e., they primarily occur within the bleb area). Therefore the local vector concentration (vg/mL) within the treated retinal tissue (i.e., MOI for cells within the bleb) is likely a better predictor for these adverse effects, compared to vg per eye (which might be more relevant for, for example, gene therapy–associated uveitis). Although the authors are confident that this is a valid approach to mechanistically probe the influence of volume on a focal adverse reaction such as retinal thinning, the fact that in this study total dose increased alongside volume must be recognized as a potential confounding factor in AAV-treated eyes.

The most important limitation of this study, however, is its overall small-scale, with only two eyes per tested volume. Because this was an exploratory study and it was initially unclear whether injection volume would have any measurable effect, the number of animals was deliberately kept limited. Once a consistent trend emerged indicating that 50 to 100 µL represented the upper tolerable limit, these findings were considered sufficient to address the primary research question (i.e., gauge the volume tolerability threshold) and further expansion of experimental or control groups was not pursued. However, the small sample size limited the potential for subgroup analyses, and the study was not sufficiently powered to explore possible synergistic interactions between volume and AAV dosage. In addition, it is important to acknowledge that the implied vulnerability to volumetric load could be outsized in the pig eye in general or even an idiosyncrasy of the *USH1C*-deficient pig retina. However, these limitations do not preclude the main finding of this study: That higher SRV correlated strongly with ONL thinning in the pig eye, whereas injection volumes of 50 µL did not cause relevant thinning, irrespective of the injected agent (BSS or AAV). This has important implications for surgeons designing preclinical trials in the pig eye, particularly when working with transgenic models. Modifying surgical technique to favor the placement of multiple smaller blebs of <100 µL, rather than a single large bleb, might mitigate retinal thinning because of volumetric stress, reduce the risk of falsely attributing retinal damage to investigational therapies, and ultimately enhance their preclinical safety profile.

## Supplementary Material

Supplement 1
