# Supplementary material for: Subretinal Injection Volume Correlates to Persistent Outer Retinal Thinning in the Pig Eye
Source: Invest Ophthalmol Vis Sci. 2025 Nov 13;66(14):27. doi: 10.1167/iovs.66.14.27 (PMC12617665; doi:10.1167/iovs.66.14.27)
Supplement: Supplement 1 [file iovs-66-14-27_s001.pdf]

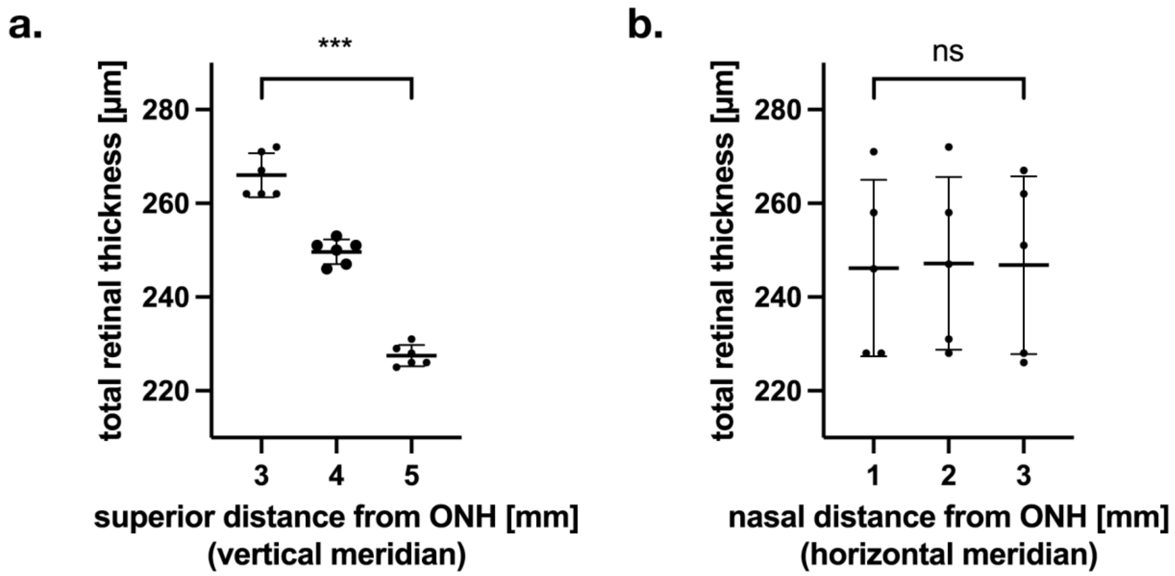

Figure S1: Total retinal thickness (TRT) along vertical and horizontal meridians in the visual streak of untreated eyes. a. Total retinal thickness in the visual streak was found to vary significantly along vertical meridians ($P<0.0001$). **b.** There was no significant variability of TRT along horizontal meridians (ns), but the absolute values were heavily dependent on the vertical position of the scan. ns: not statistically significant.

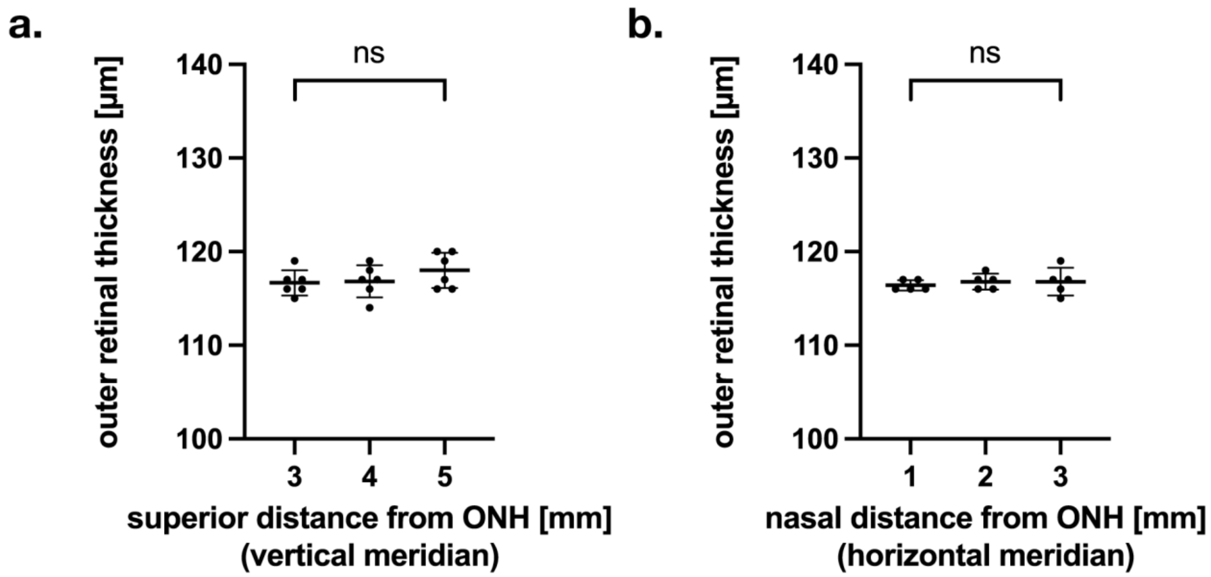

Figure S2: Outer retinal thickness (ORT) along vertical and horizontal meridians in the visual streak of untreated eyes. ORT showed a highly homogenous thickness along both **a.** vertical and **b.** horizontal meridians. ns: not statistically significant.
